# *Pseudomonas orientalis* F9: A Potent Antagonist against Phytopathogens with Phytotoxic Effect in the Apple Flower

**DOI:** 10.3389/fmicb.2018.00145

**Published:** 2018-02-09

**Authors:** Veronika Zengerer, Michael Schmid, Marco Bieri, Denise C. Müller, Mitja N. P. Remus-Emsermann, Christian H. Ahrens, Cosima Pelludat

**Affiliations:** ^1^Plant Protection Research Division, Agroscope, Zurich, Switzerland; ^2^Competence Division Methods Development, Analytics and SIB Swiss Institute of Bioinformatics, Agroscope, Zurich, Switzerland; ^3^School of Biological Sciences, University of Canterbury, Christchurch, New Zealand; ^4^Biomolecular Interaction Centre, University of Canterbury, Christchurch, New Zealand

**Keywords:** *Pseudomonas orientalis*, Erwinia amylovora, antagonistic traits, phytotoxicity, whole genome sequencing

## Abstract

In light of public concerns over the use of pesticides and antibiotics in plant protection and the subsequent selection for spread of resistant bacteria in the environment, it is inevitable to broaden our knowledge about viable alternatives, such as natural antagonists and their mode of action. The genus *Pseudomonas* is known for its metabolic versatility and genetic plasticity, encompassing pathogens as well as antagonists. We characterized strain *Pseudomonas orientalis* F9, an isolate from apple flowers in a Swiss orchard, and determined its antagonistic activity against several phytopathogenic bacteria, in particular *Erwinia amylovora*, the causal agent of fire blight. *P. orientalis* F9 displayed antagonistic activity against a broad suite of phytopathogenic bacteria in the *in vitro* tests. The promising results from this analysis led to an *ex vivo* assay with *E. amylovora* CFBP1430^Rif^ and *P. orientalis* F9 infected detached apple flowers. F9 diminished the fire blight pathogen in the flowers but also revealed phytotoxic traits. The experimental results were discussed in light of the complete genome sequence of F9, which revealed the strain to carry phenazine genes. Phenazines are known to contribute to antagonistic activity of bacterial strains against soil pathogens. When tested in the cress assay with *Pythium ultimum* as pathogen, F9 showed results comparable to the known antagonist *P. protegens* CHA0.

## Introduction

*Pseudomonas* is a diverse genus, including pathogens, tritagonists (microorganisms of unknown ecological function) and mutualists that are impacting on larger hosts (Silby et al., 2011; Freimoser et al., 2015). Members of the genus are known to synthesize an extensive number of metabolites including those that directly stimulate plant growth or that inhibit the growth of harmful microorganisms. Strains of the species *Pseudomonas fluorescens* are biocontrol agents and include *P. fluorescens* A506, and *P*. *fluorescens* EPS62e that are known for their antagonistic activity against *Erwinia amylovor*a, the causal agent of bacterial fire blight in *Rosaceae* (Wilson and Lindow, 1993; Haas and Défago, 2005; Pujol et al., 2005). As *Rosaceae* include apple and pear, fire blight is a major threat to pome fruit production worldwide causing substantial crop and economic loss. To date, the most effective control against fire blight is the application of antibiotics, e.g., streptomycin, during the flowering period. The application of antibiotics is limited due to regulatory restrictions and the rise and spread of multiple resistant pathogens (Chiou and Jones, 1995; Manulis et al., 1998; McGhee et al., 2011). In Switzerland, the application of streptomycin was banned from field applications since early 2016 and only a limited range of pest management strategies remain to hold *E. amylovora* at bay. In light of the restricted regulations and public concerns over the application of antibiotics and pesticides, the intensified use of beneficial and antagonistic organisms in agriculture is a much favorable alternative (Johnson and Stockwell, 2000; Seibold et al., 2004; Born et al., 2017).

The prerequisite for intensified biological pest management is a versatile selection of potential antagonists and an understanding of their mode of action. Some of the secondary metabolites of pseudomonads counteracting plant pathogens have already been identified. These include antibiotics, e.g., phenazines, pyrrolnitrin, pyoluteorin, and siderophores, e.g., pyoverdin, pyochelin, pseudomonin (Cornelis and Matthijs, 2002; Haas and Défago, 2005). *E. amylovora* antagonistic pseudomonads were shown to compete for space, to be more efficient in the use of nutrients or to act by cell-to-cell interference (Cabrefiga et al., 2007; Paternoster et al., 2010).

During a screen for novel antagonists against *E. amylovora*, we isolated a *P. orientalis* strain (designated F9) from apple flowers in a Swiss orchard. *P. orientalis* was first described in 1999 and isolated from Lebanese spring waters (Dabboussi et al., 1999). There is only scarce information available concerning the metabolic potential of this species, but some strains of the species that produced phenazines have been isolated from roots of dryland wheat. These isolates were associated with antagonistic activity against the Rhizoctonia root rot of wheat (Parejko et al., 2013; Jaaffar et al., 2017).

We evaluated the antagonistic activity of *P. orientalis* F9 against several plant pathogens *in vitro* and against the fire blight pathogen, *E. amylovora, in vitro* and *ex vivo*. The strain revealed antagonistic but also phytotoxic traits in the performed assays. In order to combine the experimental data with a data mining effort for genes potentially responsible for the observed traits, we sequenced *P. orientalis* F9 and *de novo* assembled the complete genome. The presence of phenazine genes prompted us to test F9 for its antagonistic activity against *Pythium ultimum* in the cress assay where F9 revealed an antagonistic activity comparable to *P. protegens* CHA0.

## Materials and Methods

### Cultivation of Bacteria and *P. ultimum*

Bacterial overnight cultures were grown at 26°C in Tryptic Soy broth (TSB, Oxoid, Karlsruhe, Germany) or King’s B medium (King et al., 1954). PSTB medium was prepared according to Pusey et al. (2008b). *P. ultimum* was cultivated on malt agar for 2–3 days (Amresco) at room temperature. Microorganisms used in this study are listed in **Table 1**.

**Table 1:** Bacterial strains used in this study.

Strain	Notes	Reference or source
*E. amylovora* CFBP 1430	Fire blight pathogen isolated from a *Crataegus* sp., 1972	Paulin and Samson, 1973
*P. orientalis* F9	Isolated from *Malus domestica*, flower, canton Zurich, CH, 2014	This study
*P. vagans* C9-1	Isolated from stem tissue of a *Malus domestica* ‘Jonathan’ in Michigan, United States. Antagonist against *E. amylovora*	Ishimaru, 1988
*P. syringae* pv. *actinidiae* ICMP 9617	Bacterial canker of kiwifruit	Collection Française de Bactéries Phytopathogènes
*P. syringae* pv. *persicae* NCPPB 2254	Bacterial die-back in peach, nectarine, Japanese plum	Collection Française de Bactéries Phytopathogènes
*P. syringae* pv. *syringae* ACW	Bacterial canker of pome and stonefruit	This study
*P. protegens* CHA0	Model organism in biological control of phytopathogens	Stutz et al., 1986
*Pythium ultimum*	Soil-born phytopathogen	Imperiali et al., 2017

### MALDI Biotyping

Matrix-assisted laser desorption/ionization (MALDI) biotyping was performed according to Gekenidis et al. (2014) using a MicroFlex biotyper, and the MALDI Biotyper software (Database Version 4.0.0.1, Bruker Daltonics Germany). Briefly, bacterial cell material from a colony on agar plate was smeared onto a MALDI target using a toothpick. The smear was then covered with 1 μl matrix solution (10 mg ml^-1^ HCCA (α-cyano-4-hydroxycinnamic acid) dissolved in acetonitrile-water-trifluoroacetic acid (TFA) (50:47.5:2.5 [vol/vol/vol]) (Sigma–Aldrich). After drying of the matrix solution, the target was placed in the MALDI biotyper and processed using the instrument’s standard settings for bacteriological classification.

### Bacterial Growth Rate Analysis

For growth rate analysis the Bioscreen C (Oy Growth Curves Ab Ltd, Helsinki) automatic microbiology growth curve analysis system was used and bacteria grown on KB plates overnight. Fresh colonies were resuspended in 1 × PBS (K_2_HPO_4_ 2.5 g l^-1^, KH_2_PO_4_ 1.2 g l^-1^) and adjusted to OD_600 nm_ = 1. Nine hundred μl medium were pipetted into a reaction, 200 μl of which were not inoculated but used as a negative control for the corresponding growth curves. The remaining 700 μl were inoculated with 3 μl of the bacterial suspension. Three replicates of the inoculated medium, each 200 μl, were loaded in wells of a Bioscreen C honeycomb plate. The plates were incubated at 26°C and shaken for 10 s before absorbance at OD_600 nm_. Absorbance was determined every 30 min for 24 h. Growth experiments were performed in two independent trials.

### Growth Inhibition Test Using the Double Layer Assay

For the double layer assay *P. orientalis* F9 and the selected plant pathogenic strains were cultivated on KB plates overnight. Fresh colonies were resuspended in 1 × PBS and adjusted to OD_600 nm_ = 1. Approximately 5 × 10^8^ bacteria were added to 10 ml of 0.75% KB top agar. Four ml of the top agar were poured onto a standard KB plate, respectively. Ten μl of the *P. orientalis* F9 suspension were spotted onto the solidified top layer surfaces. Growth halos were detected after 1–2 days of incubation at 26°C and diameter of the inhibition zones measured. The experiment was performed in duplicates.

### Siderophore Indicator Test

Siderophore production was tested on CAS (Chrome azurol S) agar, by applying 10 μl of overnight cultures of the bacterial strain to be tested. The CAS assay relies on the color change from a green-blue CAS-iron complex to orange desferrated CAS around siderophore-producing colonies (producing a CAS halo, or CAS positive) (Schwyn and Neilands, 1987).

### Biochemical Profiling of *P. orientalis* F9

For biochemical characterization of *P. orientalis* F9, Biolog GN2 and AN plates (Biolog Inc., United States) were used. The strain was cultivated overnight in 25 ml MM2 medium (4 g l^-1^ L-asparagine, 2 g l^-1^ K_2_HPO_4_, 0.2 g l^-1^ MgSO_4_, 3 g l^-1^ NaCl, 10 g l^-1^ sorbitol) at 28°C and 240 rpm. Bacteria were harvested by centrifugation at 3500 × *g* for 10 min, the cell pellet washed thrice in 1 × PBS buffer, and bacteria resuspended in 1 × PBS to an optical density of OD_600 nm_ = 0.1. This suspension was used for inoculation of the Biolog plates which were incubated for several days at 28°C and analyzed for changes of their optical density at 590 nm using a microtiter plate reader (Infinite M200, Tecan, Switzerland).

### Detached Flower Assay

For the detached flower assay (Pusey, 1997) freshly opened flowers of 2-year-old potted *Malus domestica* ‘Golden Delicious’ were used. Freshly grown colonies of *E. amylovora* CFBP1430^Rif,^ *P. vagans* C9-1 and *P. orientalis* F9 grown on KB plates were resuspended in 1 × PBS buffer to an OD_600 nm_ = 1. Twenty μl *E. amylovora* CFBP1430^Rif^ and 20 μl *P. vagans* C9-1 respectively *P. orientalis* F9 were add to 960 μl 1 × PBS. For the control inoculum, 20 μl *E. amylovora* CFBP1430^Rif^ was added to 980 μl of 1 × PBS. Twenty μl of the bacterial mixtures were directly pipetted onto the hypanthium of individual flowers. Mock treatments were performed with 1 × PBS. For each combination, 32–48 flowers were tested in two independent replicates. After inoculation, flowers were incubated at 26°C for 4–5 days. After incubation flowers were visually graded based on the following scale: grade 1: calyx green; grade 2: calyx necrotic (brownish), pedicel green; grade 3: calyx and pedicel necrotic. The severity grade was defined according to Llop et al. (2011).

### Cell Count Estimation

To estimate bacterial colony forming unit (CFU), present in single flowers, petals, pedestals, stamps, and stigmas were removed. The remains were shaken in 1 ml 1 × PBS buffer for 30 min at 1400 rpm and afterward vortexed for 30 s. A serial dilution of the bacterial suspension was performed up to 10^-7^ and 3 μl of each dilution were spotted onto TSB plates supplemented with Rifampicin (100 μg/ml) in duplicates.

### Cress Assay

For the cress assay (Rosendahl and Olson, 1992), two 1 cm diameter punched-out agar plugs of *P. ultimum* were laid on the bottom of a 9 cm diameter petri dish counterpart and carefully overlaid with 14 g twice autoclaved soil. *P. protegens* CHA0 and *P. orientalis* F9 grown on KB plates overnight, were resuspended in 1 × PBS buffer to an OD_600 nm_ = 1 followed by a 1:10 dilution. Ten ml of the diluted bacterial suspensions or the mock treatment (1 × PBS), were evenly spread over the soil surface. Subsequently, 0.4 g cress seeds (*Lepidium sativum*) were evenly distributed onto the soil. Four replicas were performed for each treatment. Plates were incubated at 22°C and 65% humidity. After 2, 4, and 6 days, 20 ml of autoclaved tap water was added. After 7 days, cress seedlings were harvested by cutting them off on ground-level and their biomass was determined by weighing. Statistical analysis was performed using the software Prism 7.0 (Graphpad Software).

### Chromosomal DNA Preparation

For chromosomal DNA isolation, cells were grown overnight in TSB and total DNA was isolated using the method of Davis (Davis et al., 1990). The quality and quantity of the extracted high molecular weight DNA was evaluated on a 0.8% (w/v) agarose gel followed by measuring absorption ratios 260 _nm_/280 _nm_ and 260 _nm_/230 _nm_ on a Nanodrop 1000 (Thermo Scientific), and by performing a Qubit dsDNA GR assay (Life Technologies, United States).

### Genome Sequencing and Assembly

Genomic DNA of *P. orientalis* F9 was sequenced on the Pacific Biosciences RS II platform and on Illumina MiSeq at the Functional Genomics Center Zurich. PacBio RS II sequencing resulted in a total of 1.03 Gbp of raw data (using two SMRT cells and P6-C4 chemistry). After quality filtering, 133,068 subreads with a mean length of 6,748 bp were obtained, which were *de novo* assembled with SMRT Portal 2.3.0 protocol *RS_HGAP_Assembly.3* (Chin et al., 2013). Terminal repeats were removed, the genome was circularized and its start position aligned with *dnaA* using Circlator 1.1.2 (Hunt et al., 2015). Remaining assembly errors were corrected by performing two rounds of resequencing (SMRT Portal 2.3.0, protocol *RS_Resequencing.1*). This resulted in one 5,986,236-bp contig of the chromosome with an average coverage depth of 130-fold. The PacBio assembly did not contain any plasmids. MiSeq sequencing was performed with a 2 × 300 bp paired end library resulting in 2.6 million read pairs. MiSeq reads were mapped to our *de novo* assembly (average coverage of 222-fold) and allowed us to detect and correct 17 single nucleotide indel errors. To capture small plasmids, which may potentially have been missed by PacBio RS II sequencing due to the size selection step during library preparation, we assembled the Illumina reads which did not map to the chromosome assembly (about 0.5% of the Illumina reads) with SPAdes 3.9 (Bankevich et al., 2012). However, no evidence for small plasmids was found. The high quality complete genome was deposited at Genbank with accession number CP018049 (Bioproject accession: PRJNA353169).

### Genome Annotation and Properties

Genome annotation was done by NCBI [Prokaryotic Genome Annotation Pipeline version 3.3 (Tatusova et al., 2016)]. COG classification was performed by searching the predicted CDSs against the eggNOG 4.5 database (Gammaproteobacteria specific dataset, “gproNOG”) (Huerta-Cepas et al., 2016) and subsequent extraction of COG categories. Hits with *e*-value higher than 0.001 were discarded. On top of the NCBI annotation, CRISPR repeats were searched with CRISPRFinder (Grissa et al., 2007) and PILER-CR (Edgar, 2007). A search for putative prophages was performed with PHASTER (Arndt et al., 2016). Genomic islands and antibiotic resistance genes within genomic islands were predicted using IslandViewer 3 (Dhillon et al., 2015). Ori-Finder (Gao and Zhang, 2008) and oriloc (Frank and Lobry, 2000) were used to extract putative positions of origin and terminus of replication. The RAST Server (Meyer et al., 2008) was used to mine specific genome properties.

### Phylogenetic Placement of *P. orientalis* F9 Based on Multilocus Sequence Analysis

For the construction of a multilocus sequence analysis-based (MLSA) phylogenetic tree (Glaeser and Kämpfer, 2015), chromosomal sequences for 12 representative *Pseudomonas* species and *Pantoea vagans* C9-1, serving as outgroup, were obtained from NCBI GenBank/RefSeq (Supplementary Table 3). Nine species of the *P. fluorescens* group including *P. orientalis* F9, two partial *P. orientalis* genome sequences, and the known *E. amylovora* antagonist *P. fluorescens* A506, were chosen. Additionally, the chromosomal sequences of less related strains *P. syringae* pv. *persicae* NCPPB 2254, *P. syringae* pv. *actinidiae* ICMP 9617, *P. syringae* pv. *syringae* ACW (representing plant pathogenic *Pseudomonas* species) but also *P. graminis* UASWS1507, and *P. citronellolis* P3B5 were added to cover a broader phylogenetic spectrum (Takikawa et al., 1989; Remus-Emsermann et al., 2016). The nucleotide sequences of the housekeeping genes *gyrB, recA, rpoB* and *rpoD* were extracted from all genome sequences, respectively. Multiple sequence alignment (MSA) using MUSCLE 3.8 (Edgar, 2004) was performed on the individual genes to ensure that the extracted sequences were suitable for phylogenetic analysis. The nucleotide sequences of the four genes were concatenated for every strain and a final MUSCLE MSA was performed. With the resulting alignment of 9,520 nucleotides length, a maximum likelihood based phylogenetic inference was performed using RAxML 8.1 (Stamatakis, 2014).

## Results

### *P. orientalis* F9 Is a Member of the Fluorescent *Pseudomonas* Group

*Pseudomonas orientalis* F9 was isolated in spring 2014 from Boskoop apple flowers in a Swiss orchard during a screen for novel bacterial biocontrol agents of *E. amylovora*. F9 was classified as *P. orientalis* using MALDI biotyping with a moderate score. *P. orientalis* is a member of the *P. fluorescens* group within the genus (Dabboussi et al., 1999). The fluorescence is due to the synthesis of siderophores, iron chelators that are secreted under low iron conditions (Neilands, 1995). Indeed, isolate F9 exhibited a halo on CAS agar, which is indicative for siderophore production, and showed fluorescence when cultivated under low iron conditions (data not shown).

### Genome Properties of *P. orientalis* F9

**FIGURE 1:**
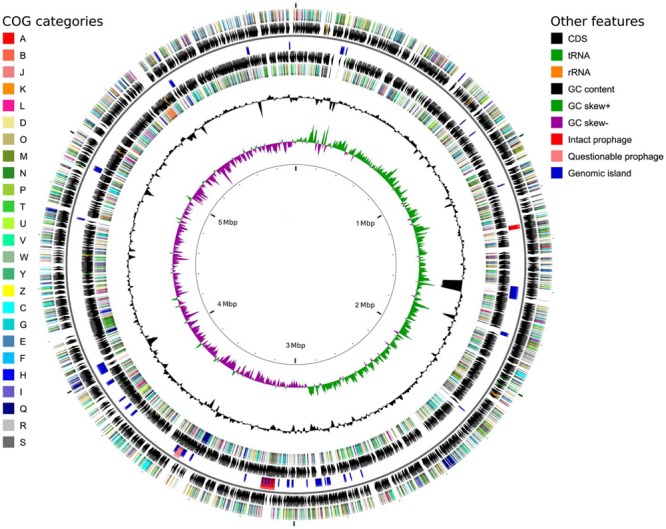
Circular genome map for *P. orientalis* F9, generated using CGView (Stothard and Wishart, 2005). The following features are shown (moving from the outermost track inward, origin of replication at 0 Mbp): (1) CDS on forward strand colored according to COG category, (2) CDS (*black*), tRNA (*green*) and rRNA (*orange*) on forward strand, (3) Intact prophages (*red*), questionable prophages (*light red*) and genomic islands (*blue*) for both strands, (4) CDS (*black*), tRNA (*green*) and rRNA (*orange*) on reverse strand, (5) CDS on reverse strand colored according to COG category, (6) GC content (*black*), (7) positive and negative GC skew (*green* and *purple*, respectively) and (8) genome position in Mbp.

To verify the identification as a *P. orientalis* isolate and to further analyze its genome properties, strain F9 was sequenced and *de novo* assembled into a closed, high quality complete genome using a hybrid strategy (Koren et al., 2012), i.e., relying on PacBio long reads and Illumina MiSeq short reads (see Methods). No plasmids were detected. The 5.99 Mbp genome has an average GC content of 60.4%. Of the 5,271 predicted genes, 5,142 (97.6%) were predicted as protein coding sequences (CDS), 19 as rRNAs (0.4%), 67 as tRNAs (1.3%), 4 as ncRNAs (0.1%) and 39 as pseudogenes (0.7%) (**Table 2**). **Figure 1** shows a circular view of the genome. The GC content deviates from the mean value in regions where prophages, genomic islands or rDNA clusters are located. The GC skew splits up at the putative origin of replication [confirmed using Ori-Finder (Gao and Zhang, 2008) and oriloc (Frank and Lobry, 2000)] and the putative terminus of replication (confirmed by oriloc). This provided additional evidence for the correctness of the assembly. PHASTER (Arndt et al., 2016) detected two intact prophages (Locus tags BOP93_05630 to BOP93_05745 and BOP93_13270 to BOP93_13665) and one questionable prophage (Locus tags BOP93_15105 to BOP93_15245) (**Table 2**), while IslandViewer 3 (Dhillon et al., 2015) detected a large number of genomic islands (53, positions on the genome provided in Supplementary Table 4). Information about the COG classification of all CDSs can be found in Supplementary Table 1. A brief comparison of the two already available *P. orientalis* genome assemblies (strains BS2775 and DSM 17489) versus our complete *de novo* assembly can be found in Supplementary Table 2.

**Table 2:** Genome statistics.

Attribute	Value	Percentage
Genome size (bp)	5,986,236	100.0%
DNA coding (bp)	5,182,176	86.6%
DNA G + C (bp)	3,614,136	60.4%
Total number of genes	5,271	100.0%
Protein coding genes (CDS)	5,142	97.6%
rRNA genes	(5S/16S/23S → 7/6/6) 19	0.4%
tRNA genes	67	1.3%
ncRNA genes	4	0.1%
Pseudogenes	39	0.7%
Genes with function prediction (NCBI annotation pipeline, see Methods)	4,154	78.8%
CDS assigned to COG class	4,699	
CRISPR repeats/Cas Proteins	0/0 #	
Intact prophages ^∗^	2	
Questionable prophages ^∗^	1	

*^∗^Classification used by PHASTER (Arndt et al., 2016). #Neither CRISPR repeats nor Cas proteins were found, hence F9 seems to lack a CRISPR/Cas system.*

### Phylogenetic Placement of *P. orientalis* F9

**FIGURE 2:**
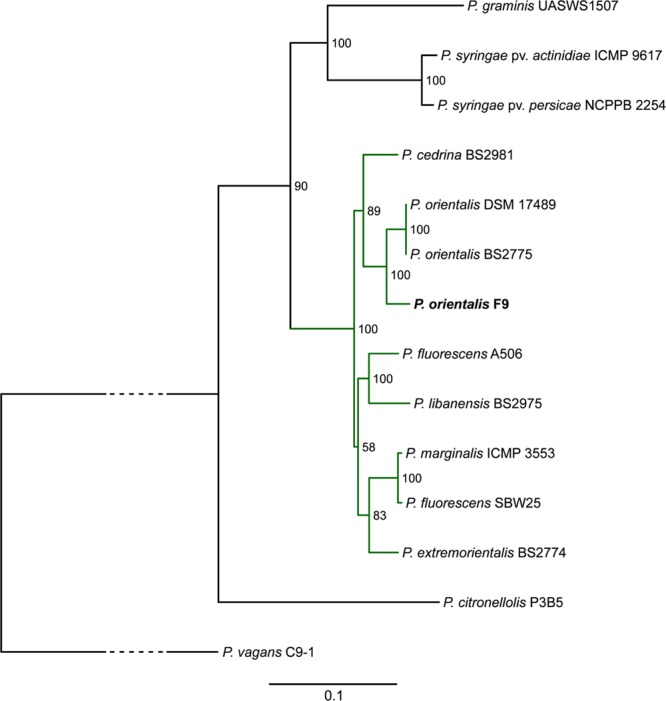
Phylogenetic tree highlighting the position of *P. orientalis* F9 (bold) relative to other representative *Pseudomonas* species (green subtree is representing the *P. fluorescens* group). The tree is based on MLSA of the four housekeeping genes *gyrB, recA, rpoB*, and *rpoD*. Maximum likelihood based phylogenetic inference was performed with RAxML. Numbers at the branches indicate the percentage of replicate trees in which associated taxa clustered in the bootstrap test (100 replicates). *P. vagans* C9-1 served as outgroup; the bottom bar reflects the estimated number of nucleotide changes per site between two nodes in the tree. The dashed lines represent regions of the phylogenetic tree where the branches were collapsed. Accession numbers of the strains are given in Supplementary Table 3.

A MLSA phylogenetic tree based on the housekeeping genes *gyrB, recA, rpoB*, and *rpoD* was calculated to compare the placement of *P. orientalis* F9 with twelve representative *Pseudomonas* species and *Pantoea vagans* C9-1 (outgroup) phylogenetically. In accordance with MALDI biotyping, the resulting tree places *P. orientalis* F9 closely to two other *P. orientalis* strains (**Figure 2**) within the group of the fluorescent pseudomonads. The phylogeny presented in the phylogenetic tree is in accordance with a recent phylogenomics and systematics study by Gomila et al. (2015) although a detailed comparison is difficult since the taxonomy of many *Pseudomonas* type strains is outdated.

### Biochemical Profile and Growth of *P. orientalis* F9 in PSTB and KB Medium

**FIGURE 3:**
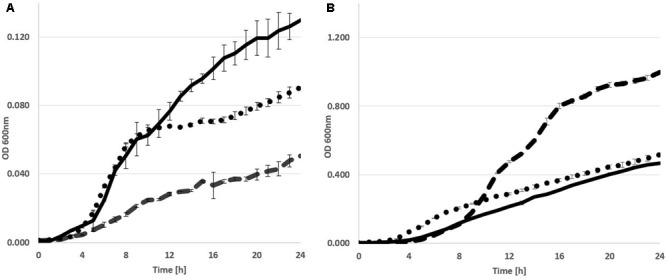
Growth curve of *E. amylovora* CFBP1430 ^Rif^ (Ea CFBP1430, black line), *P. vagans* C9-1 (Pv C9-1, dotted line), and *P. orientalis* F9 (Po F9, broken line), in PSTB **(A)** or KB medium **(B)**. Measurement of OD (A_600 nm_) was performed every 30 min for 24 h. Error bars represent standard deviations. Area under curve in PSTB: Ea CFBP1430 = 1.68 (±0.02); Pv C9-1 = 1.3 (±0.005); Po F9 = 0.59 (±0.007) in KB: Ea CFBP1430 = 5.19 (±0.015); Pv C9-1 = 6.5 (±0.033); Po F9 = 11.2 (±0.033).

The metabolic versatility of *P. orientalis* F9 with regard to the ability to metabolize major components present on the stigma was assessed using Biolog GN2, AN2 and GenIII plates. The strain was able to utilize L-asparagine, L-aspartic acid, L-proline, D-sorbitol but not D-fructose. As *P. orientalis* F9 was isolated from apple flowers, we evaluated its growth in PSTB medium that mimics the nutrient composition on stigma (Pusey et al., 2008a). *P. orientalis* F9’s growth in PSTB was compared to that of *E. amylovora* CFBP1430^Rif^ and *P. vagans* C9-1 (**Figure 3**). F9 was growing in the sugar-rich PSTB medium (max growth rate μ = 0.33) but slower than both the fire blight pathogen (μ = 0.39) and *P. vagans* C9-1 (μ = 0.41). In contrast, *P. orientalis* F9 grew faster (μ = 0.49) than both strains (CFBP1430^Rif^: μ = 0.31, C9-1: μ = 0.41) in iron limited KB medium. Due to the ability of F9 to efficiently grow in iron deficient medium, we tested its potential as biocontrol agent under iron limited conditions.

### *P. orientalis* F9 Induces Growth Deficiency of *E. amylovora* CFBP1430^Rif^ and *P. syringae* Pathovars *in Vitro*

When analyzing the *P. orientalis* F9 genome for potential siderophore synthesis genes, genes related to pyoverdine production were detected (CDSs BOP93_10400 to BOP93_10440). Pyoverdine is the generic name given to a vast family of fluorescent green-yellowish pigments produced by *Pseudomonas* species and represents their primary siderophore (Cornelis and Matthijs, 2002; Cornelis, 2010).

**FIGURE 4:**
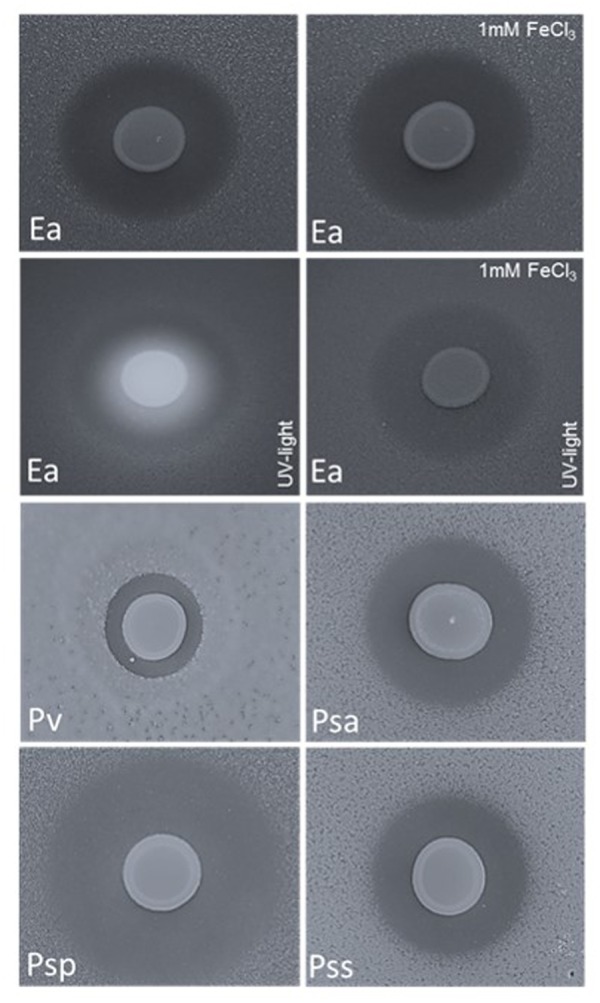
Double layer assay with *E. amylovora* CFBP1430^Rif^ (Ea); *P. vagans* C9-1 (Pv, halo size 0.3 cm); *P. syringae* pv. *actinidiae* ICMP 9617 (Psa, halo size 0.7 cm); *P. syringae* pv. *persicae* NCPPB 2254 (Psp, halo size 1.2 cm) and *P. syringae* pv. *syringae* ACW (Pss, halo size 0.6) seeded in the top layer and *P. orientalis* F9 applied onto the surface (10 μl of a *P. orientalis* F9 suspension of OD_600 nm_ = 1). Application of Fe^3+^ (1 mM FeCl_3_) to the medium had no impact on the growth halo formed by the fire blight pathogen but abolished the fluorescence that appears concomitantly with siderophore production.

The impact of pyoverdine on the growth of *E. amylovora* CFBP1430 *in vitro* was analyzed in a double layer assay. The assay was performed with iron limited KB medium and *E. amylovora* CFBP1430^Rif^ seeded in the top layer. Application of *P. orientalis* F9 onto the surface of the top layer led to a clear growth halo of *E. amylovora* CFBP1430^Rif^, that could not be abrogated by addition of FeCl_3_ (**Figure 4**). To test if *P. orientalis* F9 affects the growth of additional bacteria, plant pathogenic strains *P. syringae* pv. *syringae* ACW (causing bacterial canker of pome and stonefruit, *P. syringae* pv. *actinidiae* ICMP 9617 [bacterial canker of kiwifruit (Takikawa et al., 1989)], and *P. syringae* pv. *persicae* NCPPB 2254 (bacterial die-back in peach, nectarine, Japanese plum) and also *P. vagans* C9-1, were added to the assay (Paulin and Samson, 1973; Ishimaru, 1988; Young, 1988; Takikawa et al., 1989).

*P. orientalis* F9 induced halos in all tested strains after 1–2 days of incubation (**Figure 4**). Again, addition of iron to the medium had no visible impact on halo formation or halo size (data not shown) implying that iron deprivation caused by pyoverdine synthesis of *P. orientalis* F9 is not the basic cause for growth deficiency of the tested strains.

### The Genome of *P. orientalis* F9 Harbors the Safracin and Phenazine Clusters

We mined the *P. orientalis* F9 genome to address the question of potential mode of actions for the demonstrated antagonistic activity and could identify several potential candidate genes: (i) *P. orientalis* F9 carries a safracin cluster (Locus tags BOP93_17395 to BOP93_17440) that is almost 100% identical to the cluster present in *P. fluorescens* A2-2 (Accession: AY061859.1). Safracin is a compound with potent broad-spectrum antibacterial activities (Velasco et al., 2005). (ii) F9 harbors, the *phzABCDEFG* operon (Locus tags BOP93_12290 to BOP93_12320). Phenazine production of *P. orientalis* strains could also be observed in case of isolates from dryland wheat (Parejko et al., 2013). All phenazine-producing bacteria contain orthologs of this core biosynthesis operon which is required for the conversion of chorismic acid into the broad-spectrum antibiotic phenazine-1-carboxylate. Phenazines are a diverse group of secondary metabolites with broad-spectrum antibiotic activity against bacteria, fungi, and eukaryotes. They are actively involved in the suppression of plant pathogens and phenazines produced by *P. agglomerans* on apple flowers contribute to the suppression of *E. amylovora*, (Thomashow et al., 1990; Giddens et al., 2003). Furthermore, genes *phzI* (BOP93_12330) and *phzR* (BOP93_12325) that encode a quorum-sensing circuit which regulates the phenazine production (Pierson et al., 1994) are located upstream of the *phz*-operon.

### *P. orientalis* F9 Proliferates in Apple Flowers

*P. orientalis* F9 was isolated from apple flowers. To confirm its ability to successfully proliferate in apple flowers the strain was inoculated at low densities (20–70 CFU) onto the hypanthium. It reached population densities of 10^7^ CFU per flower after 5 days of incubation, a value comparable to that reported for other flower colonizing bacteria (Cabrefiga et al., 2007).

### *P. orientalis* F9 Shows Ambiguous Results in the “Detached Flower Assay”

**FIGURE 5:**
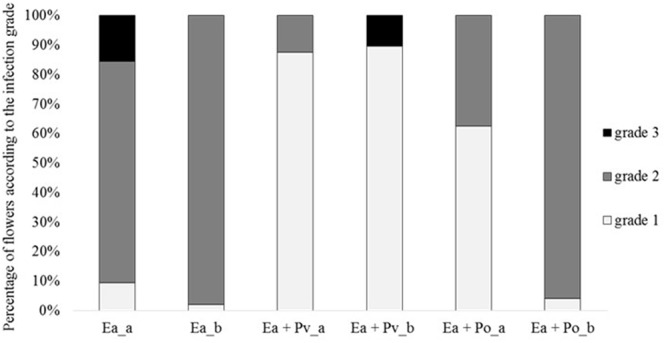
Percentage of flowers according to the infection grade of two independent detached flower assay experiments (a and b). Each column represents 32 (a) or 48 (b) flowers. Flowers were inoculated with *E. amylovora* CFBP1430^Rif^ (Ea), *E. amylovora* CFBP1430^Rif^ co-inoculated with *P. vagans* C9-1 (Ea + Pv) or *E. amylovora* CFBP1430^Rif^ co-inoculated with *P. orientalis* F9 (Ea + Po). Evaluation of the flowers were performed according to the following scale: grade 1: calyx green; grade 2: calyx necrotic; grade 3: calyx and pedicel necrotic.

As *P. orientalis* F9 has the ability to proliferate in apple flowers and induces growth deficiency of *E. amylovora* CFBP1430^Rif^ *in vitro*, its antagonistic activity was tested *ex vivo* using the “detached flower assay” (Pusey, 1997). In two independent experiments detached apple flowers (24 each) were inoculated with binary strain mixtures, combining *E. amylovora* CFBP1430^Rif^ with either the well-described antagonist *P. vagans* C9-1 or *P. orientalis* F9, respectively. As a control, the fire blight pathogen was inoculated as single culture. After 4–5 days of incubation the flowers were visually rated (**Figure 5**). In both trials, less than 10% of the flowers infected with the pathogen only were evaluated with grade 1 (indicative for a phenotypically healthy flower). The severity of infection for the control was 69 (Ea_a) in the first - and 66 (Ea_b) in the second trial. When co-inoculated with *P. vagans* C9-1, a significant increase of healthy flowers was observed (severity Ea + Pv_a = 38, Ea + Pv_b = 40). Flowers inoculated with *P. orientalis* F9 and *E. amylovora* CFBP1430^Rif^ yielded ambiguous results between the trials. Even though an increase of healthy flowers (ca. 60%) could be detected in the first trial (severity Ea + Po_a = 46), there was no difference to the *E. amylovora* CFBP1430^Rif^ control infection in the second (severity Ea + Po_b = 65).

### Co-inoculation with *P. orientalis* F9 Diminishes *E. amylovora* CFBP1430^Rif^ CFU in Apple Flowers

**FIGURE 6:**
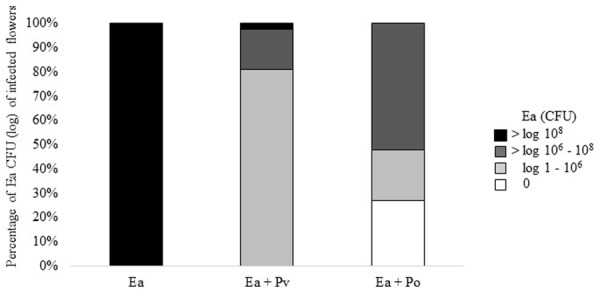
Percentage of log CFU and 0 CFU of *E. amylovora* CFBP1430^Rif^ recovered from infected apple flowers. Forty eight flowers (=100%) were inoculated with *E. amylovora* CFBP1430^Rif^ (Ea), *E. amylovora* CFBP1430^Rif^ co-inoculated with *P. vagans* C9-1 (Ea + Pv) or *E. amylovora* CFBP1430^Rif^ co-inoculated with *P. orientalis* F9 (Ea + Po).

Results of the second trial prompted us to test infected flowers for the presence of *E. amylovora*. Bacteria were re-isolated from flowers and plated onto rifampicin containing TSB agar to select for *E. amylovora* CFBP1430^Rif^. Flowers infected with only *E. amylovora*, consistently carried > 10^7^ CFU. Co-inoculation with *P. vagans* C9-1 decreased the number of *E. amylovora* CFBP1430^Rif^ recovered from flowers, 70% of the flowers carried 10^5^ to 10^7^ *E. amylovora*. Co-inoculation of *E. amylovora* CFBP1430^Rif^ with *P. orientalis* F9 resulted in the lowest numbers of the fire blight pathogen (**Figure 6**). In 13 apple flowers (27%) no pathogen could be detected. Whilst having the lowest estimated CFU of the pathogen, the visual rating of the *E. amylovora* CFBP1430^Rif^/*P. orientalis* F9 infection was comparable to that of the infection control. This discrepancy points to a phytotoxic potential of *P. orientalis* F9 in apple flowers.

### *P. orientalis* F9 Reveals Phytotoxic Traits in Detached Apple Flowers

**FIGURE 7:**
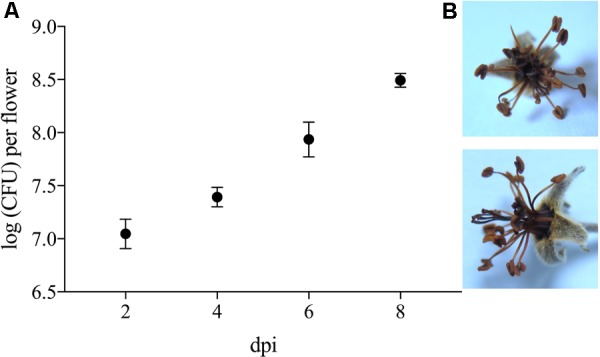
**(A)** Log CFU of *P. orientalis* F9 recovered from detached apple blossom on day two, four, six and eight post infection. Error bars represent the standard error of the mean. **(B)** Necrotic flower 6 days post infection.

To evaluate its phytotoxic traits, *P. orientalis* F9 was inoculated (ca. 5 × 10^5^) onto the hypanthium of apple flowers. Two, four, six, and eight days post infection (dpi), flowers were visually evaluated and the CFU of *P. orientalis* F9 determined. After day six, half of the flowers showed a necrotic phenotype (**Figure 7**). The increase of flowers necrosis and CFU correlated, implying the degradation of flowers to be an additional nutrient source for the strain. When mining the F9 genome for *P. syringae* related phytotoxins (syringomycin, phaseolotoxin, syringopeptin, tabtoxin) as source for the observed phenotype, their presence could not be confirmed.

### *P. orientalis* F9 Exhibits a Protective Phenotype in the Cress Assay

**FIGURE 8:**
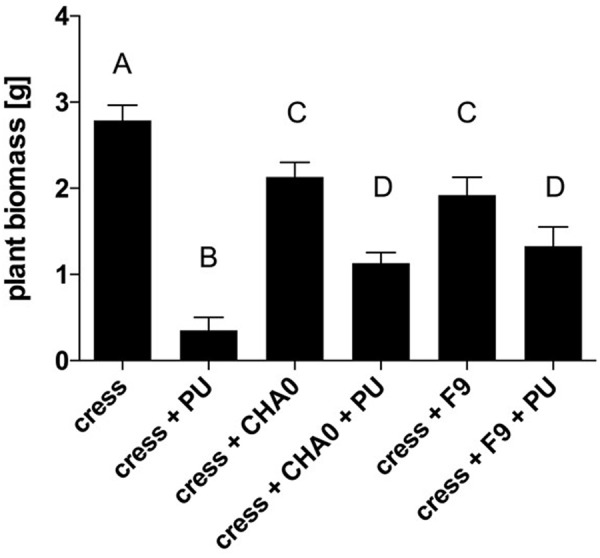
Cress biomass after treatment with *P. ultimum* (PU), *P. protegens* CHA0 (CHA0), and/or *P. orientalis* F9 (Po). Cress biomass was harvested 7 days post inoculation. Error bars represent standard error of the means. Bars with the same letter represent results that are not significantly different (*P* > 0.05, one-way ANOVA with Tukey’s multiple comparisons test).

Genome mining revealed that *P. orientalis* F9 is possessing the phenazine cluster, which is also present in pseudomonas strains that antagonize wheat pathogens (Jaaffar et al., 2017). To evaluate the potential of F9 as antagonist of soil-borne pathogens we used the cress assay (Rosendahl and Olson, 1992). The oomycete *P. ultimum* was used as a model soil pathogen and *P. protegens* CHA0 served as a reference strain for antagonistic activity. CHA0 is a biocontrol agent that protects cucumber from several fungal pathogens, including *Pythium* spp. (Keel et al., 1992) and is often used as model strain in biocontrol experiments. Results of the cress assay showed that after infection with *P. ultimum* the average cress biomass was 0.35 g per petri dish. In presence of *P. ultimum* and *P. protegens* CHA0 the biomass increased to 1.13 g on average. When treated with *P. ultimum* and *P. orientalis* F9, the biomass accumulated to 1.32 g (**Figure 8**). Both pseudomonads have an effect on cress weight when compared to mock treated controls cress only. There is no evidence that this is due to the phytotoxic effect that F9 exhibits on blossoms, as both strains have the effect (**Figure 8**).

## Discussion

In light of public concerns over the use of antibiotics and also pesticides in plant protection, effective biological control in form of antagonistic bacteria should be considered as an alternative in fire blight management. A prerequisite for the successful application of antagonists with high biocontrol efficacy and stability is a broad set of well-defined microorganisms with antagonistic activity and an understanding of their modes of action. This would allow to choose optimal antagonists for application under a wide range of given environmental conditions and diseases. Pseudomonads, known for their metabolic versatility and genetic plasticity, encompass plant pathogens but also antagonists that are being applied against plant pathogens including *E. amylovora*, the cause of fire blight (Wilson and Lindow, 1993; Galasso et al., 2002; Paternoster et al., 2010; Mikiciński et al., 2016).

We isolated bacterial strain F9, from apple flowers in a Swiss orchard. F9 was classified by MALDI-TOF biotyping as *P. orientalis*. We sequenced and *de novo* assembled the complete genome of *P. orientalis* F9 using a hybrid approach of PacBio long reads and Illumina MiSeq data, providing the basis for accurate and comprehensive genome annotation (Omasits et al., 2017). The assembly of the reads revealed a 5.99 Mbp genome with an average GC content of 60.4% and 5,142 predicted protein coding genes. A MLSA with four housekeeping genes of both strain F9 and twelve related *Pseudomonas* species strains confirmed F9 to be a *P. orientalis* strain. To the best of our knowledge, the here presented F9 genome represents the first complete and circularized full genome sequence of a *P. orientalis* strain (Supplementary Table 2).

*Pseudomonas orientalis* was first described in Dabboussi et al. (1999); but even to date an in-depth characterization of the species is lacking. We tested *P. orientalis* F9 for its antagonistic activity against *E. amylovora* CFBP 1430. The pathogen grows epiphytically on stigmata before it enters through the nectaries from where it spreads into the tissue, resulting in flower and shoot blight symptoms (Thomson, 2000). Current control efforts focus on the limitation of the pathogen in the flower. The application of antibiotics (e.g., streptomycin) is thus far the most successful control measure against fire blight, however, it is also controversially discussed, due to concerns about possible antibiotic resistance spread (Jones and Schnabel, 2000; McManus et al., 2002). *In vitro* the strain successfully antagonized the fire blight pathogen in the KB double layer assay. In addition, F9 induced growth deficiency not only in *E. amylovora* CFBP1430, but also in several tested *P. syringae* pathovar strains and in *P. vagans* C9-1, an *E. amylovora* antagonist (**Figure 4**). We tested if F9‘s mode of action in the assay is based on siderophore production as KB is an iron deficient medium. Under iron limiting conditions bacteria secrete siderophores, high affinity iron chelators that bind Fe^3+^ and thereby make it available for the cell (Braun and Winkelmann, 1987; Neilands, 1995). *E. amylovora* harbors one siderophore system; the hydroxamate siderophore desferrioxamine E (DFO E). The importance of this system for the pathogenicity of *E. amylovora* has been demonstrated by siderophore synthesis or uptake mutants that exhibit decreased pathogenicity on apple flowers (Dellagi et al., 1998).

Pseudomonads are known for their large repertoire of siderophores and pyoverdines are the main siderophores produced by fluorescent pseudomonads. F9 exhibits strong fluorescence when cultivated on KB medium and indeed, the strain harbors pyoverdine synthesis genes in the genome (Cornelis and Matthijs, 2002). However, as the induced growth halos neither disappeared nor were reduced in size after addition of iron to the medium, siderophore production of F9 and subsequent iron deprivation of the tested strains are unlikely to be the cause for growth inhibition. An alternative explanation for the ability of F9 to serious growth deficiencies in strains belonging to different genera is given by the biosynthesis genes of phenazine and safracin that are located on the chromosome. Both components have anti-bacterial activity (Velasco et al., 2005). Phenazines are known to antagonize plant pathogens especially in the rhizosphere but also in the apple flower (Giddens et al., 2003). For *Pantoea* strains such as *P. agglomerans* Eh325, *P. agglomerans* Eh318 or *P. vagans* C9-1, the antagonistic effect against the fire blight pathogen is mainly attributed to the production of antibiotics (Wright et al., 2001; Stockwell et al., 2002; Pusey et al., 2011; Kamber et al., 2012). In case of *P. agglomerans* Eh325 the antibiotic is even specific for *E. amylovora* (Pusey et al., 2008b) which is not true for the F9 antibiotics that exhibited a broad host range and effected all bacteria tested. *P. synxantha* 2-79 is known for its antagonistic activity against the fungus *Gaeumannomyces graminis* var. *tritici*, a major root disease of wheat. *P. synxantha* 2–79 produces phenazine and pyoverdin. Pyoverdin mutants of *P. synxantha* 2–79 controlled the pathogen as effectively as the fluorescent parental strains, revealing the phenazine antibiotic to be the dominant factor in the antagonistic activity of the strain (Hamdan et al., 1991).

F9’s promising antagonistic traits in the *in vitro* double layer assay prompted us to test for its antagonistic activity against the fire blight pathogen *ex vivo.* Before performing the “detached flower assay” the growth of *P. orientalis* F9 was tested in detached apple flowers. The strain not only persisted but successfully proliferated in apple flowers and reached up to 10^7^ CFU. In the following two independent trials *P. orientalis* F9 was co-inoculated with *E. amylovora* CFBP1430^Rif^ in detached apple flowers. While an increase of healthy flowers (ca. 60%) could be detected in the first trial, there was no difference to the *E. amylovora* CFBP1430^Rif^ control infection in the second (**Figure 5**). In contrast to the visual rating, the CFU of *E. amylovora* CFBP1430^Rif^ in apple flowers co-inoculated with *P. orientalis* F9 flowers was lower than in the control with 13 apple flowers (27%) free of the pathogen. The discrepancy between visual rating and CFU of *E. amylovora* in the detached flower assay can be explained by phytotoxicity of *P. orientalis* F9 in apple flowers that could be confirmed by inoculation of flowers with F9 only. Flower necrosis and increase of F9 CFU correlated. Necrosis was only detectable when the initial inoculate of the strain was approximately 10^6^ CFU. Flowers inoculated with less than 10^2^ bacteria showed no necrotic symptoms after 5 dpi and presence of *P. syringae* related phytotoxins (syringomycin, phaseolotoxin, syringopeptin, tabtoxin) on the F9 chromosome could not be confirmed. A recent publication showed that *P. orientalis* strains may be pathogenic to citrus species after leaf infiltration (Beiki et al., 2016). Here, we provide further evidence that *P. orientalis* may act as a plant pathogen, however, we propose that on plant it is an opportunistic pathogen rather than a *bona fide* disease agent. Data mining of the *P. orientalis* F9 genome, implied F9 to be capable of producing phenazines. Phenazines producing *P. orientalis* strains successfully antagonize wheat pathogens (Jaaffar et al., 2017). Thus, the ability of F9 as protective agent against soil-born pathogens was tested in the cress assay. Indeed, when compared to the extensively studied antagonist *P. protegens* CHA0, *P. orientalis* F9 protected cress to a comparable degree (**Figure 8**). The antagonistic activity of *P. protegens* CHA0 is not attributed to phenazines but mainly to the production of the antimicrobial polyketides 2,4-diacetylphloroglucinol and pyoluteorin. *P. orientalis* F9 is as efficient as CHA0 in the cress assay. It has to be elucidated, if these antagonistic traits are effective in longer term protection experiments and which genes are responsible for the exhibited traits. In the presented paper genome mining was the tool that led to the shift in antagonistic studies of a bacterial isolate from phyllosphere to rhizosphere with promising results. Genome comparison of F9 to additional *P. orientalis* and *Pseudomonas* strains will be an important tool to understand and characterize pseudomonads and their mode of actions. In the future, it might reveal additional factors that specify the environment in which F9 evolve its optimal potential as an antagonist.

MALDI-TOF biotyping and genome sequencing are modern techniques that facilitate the selection of antagonists and may ultimately provide the basis to unravel their mode of action. MALDI-TOF is a fast tool to eliminate phytopathogenic or restricted bacteria from the antagonist screening process. Annotated complete genome sequences of potential and tested antagonists can provide a first indication of underlying mechanisms and of the genes involved by e.g., applying principles of gene trait matching. Further validations, e.g., constructing mutants of the *in silico* predicted genes can confirm their significance in the antagonistic activity of bacteria. Future approaches in the evaluation process of potential antagonists will involve genome mining complemented with gene expression analysis under different conditions (Omasits et al., 2013). This will not only provide important information to decipher genes and pathways active under the given conditions but also potential mechanisms underlying the antagonistic effects, criteria that can be used for an improved strain selection.

## Author Contributions

VZ isolated the strain, performed detached flower assays, inhibition assays, MALDI, and determined the CFU. MS planned and performed the bioinformatic analysis, assembled and annotated the genome, performed phylogenetic analysis and contributed to the writing of the manuscript. MB performed studies on the proliferation of F9 in the apple flowers. DM performed the cress assay and critically read the manuscript. MR-E performed and analyzed the biochemical characterization, analyzed data and wrote the manuscript. CA planned and supervised the bioinformatic analysis, and contributed to the writing of the manuscript. CP conceived the study, isolated DNA, performed the CAS assay and wrote the manuscript. All authors critically read and approved the final manuscript.

## Conflict of Interest Statement

The authors declare that the research was conducted in the absence of any commercial or financial relationships that could be construed as a potential conflict of interest.

## References

[B1] Arndt D. Grant J. R. Marcu A. Sajed T. Pon A. Liang Y. (2016). PHASTER: a better, faster version of the PHAST phage search tool. *Nucleic Acids Res.* 44 W16–W21. 10.1093/nar/gkw387 27141966 PMC4987931

[B2] Bankevich A. Nurk S. Antipov D. Gurevich A. A. Dvorkin M. Kulikov A. S. (2012). SPAdes: a new genome assembly algorithm and its applications to single-cell sequencing. *J. Comput. Biol.* 19 455–477. 10.1089/cmb.2012.0021 22506599 PMC3342519

[B3] Beiki F. Busquets A. Gomila M. Rahimian H. Lalucat J. García-Valdés E. (2016). New *Pseudomonas* spp. are pathogenic to citrus. *PLOS ONE* 11:e0148796. 10.1371/journal.pone.0148796 26919540 PMC4769151

[B4] Born Y. Fieseler L. Thöny V. Leimer N. Duffy B. Loessner M. J. (2017). Engineering of bacteriophages Y2::dpoL1-C and Y2::luxAB for efficient control and rapid detection of the fire blight pathogen, *Erwinia amylovora*. *Appl. Environ. Microbiol.* 83 e00341-17. 10.1128/AEM.00341-17 28389547 PMC5452800

[B5] Braun V. Winkelmann G. (1987). Microbial iron transport structure and function of siderophores. *Prog. Clin. Biochem. Med.* 5 67–99. 10.1007/978-3-642-72902-7_4

[B6] Cabrefiga J. Bonaterra A. Montesinos E. (2007). Mechanisms of antagonism of *Pseudomonas fluorescens* EPS62e against *Erwinia amylovora*, the causal agent of fire blight. *Int. Microbiol.* 10 123–132. 17661291

[B7] Chin C.-S. Alexander D. H. Marks P. Klammer A. A. Drake J. Heiner C. (2013). Nonhybrid, finished microbial genome assemblies from long-read SMRT sequencing data. *Nat. Methods* 10 563–569. 10.1038/nmeth.2474 23644548

[B8] Chiou C. S. Jones A. L. (1995). Expression and identification of the strA-strB gene pair from streptomycin-resistant *Erwinia amylovora*. *Gene* 152 47–51. 10.1016/0378-1119(94)00721-4 7828927

[B9] Cornelis P. (2010). Iron uptake and metabolism in pseudomonads. *Appl. Microbiol. Biotechnol.* 86 1637–1645. 10.1007/s00253-010-2550-2 20352420

[B10] Cornelis P. Matthijs S. (2002). Diversity of siderophore-mediated iron uptake systems in fluorescent pseudomonads: not only pyoverdines. *Environ. Microbiol.* 4 787–798. 10.1046/j.1462-2920.2002.00369.x 12534462

[B11] Dabboussi F. Hamze M. Elomari M. Verhille S. Baida N. Izard D. (1999). Taxonomic study of bacteria isolated from Lebanese spring waters: proposal for *Pseudomonas cedrella* sp.nov. and *P. orientalis* sp. nov. *Res. Microbiol.* 150 303–316. 10.1016/S0923-2508(99)80056-4 10422691

[B12] Davis L. G. Dibner M. D. Battley J. F. (1990). *Basic Methods in Molecular Biology.* New York, NY: Elsevier.

[B13] Dellagi A. Brisset M. N. Paulin J. P. Expert D. (1998). Dual role of desferrioxamine in *Erwinia amylovora* pathogenicity. *Mol. Plant Microbe Interact.* 11 734–742. 10.1094/MPMI.1998.11.8.734 9675889

[B14] Dhillon B. K. Laird M. R. Shay J. A. Winsor G. L. Lo R. Nizam F. (2015). IslandViewer 3: more flexible, interactive genomic island discovery, visualization and analysis. *Nucleic Acids Res.* 43 W104–W108. 10.1093/nar/gkv401 25916842 PMC4489224

[B15] Edgar R. C. (2004). MUSCLE: a multiple sequence alignment method with reduced time and space complexity. *BMC Bioinformatics* 5:113. 10.1186/1471-2105-5-113 15318951 PMC517706

[B16] Edgar R. C. (2007). PILER-CR: fast and accurate identification of CRISPR repeats. *BMC Bioinformatics* 8:18. 10.1186/1471-2105-8-18 17239253 PMC1790904

[B17] Frank A. C. Lobry J. R. (2000). Oriloc: prediction of replication boundaries in unannotated bacterial chromosomes. *Bioinformatics* 16 560–561. 10.1093/bioinformatics/16.6.560 10980154

[B18] Freimoser F. M. Pelludat C. Remus-Emsermann M. N. P. (2015). Tritagonist as a new term for uncharacterised microorganisms in environmental systems. *ISME J.* 10 1–3. 10.1038/ismej.2015.92 26035056 PMC4681867

[B19] Galasso O. Sponza G. Bazzi C. Vanneste J. L. (2002). Characterization of two fluorescent strains of *Pseudomonas* as biocontrol agents against fire blight. *Acta Hortic.* 590 299–307. 10.17660/ActaHortic.2002.590.44

[B20] Gao F. Zhang C.-T. (2008). Ori-Finder: a web-based system for finding oriCs in unannotated bacterial genomes. *BMC Bioinformatics* 9:79. 10.1186/1471-2105-9-79 18237442 PMC2275245

[B21] Gekenidis M.-T. Studer P. Wüthrich S. Brunisholz R. Drissner D. (2014). Beyond the matrix-assisted laser desorption ionization (MALDI) biotyping workflow: in search of microorganism-specific tryptic peptides enabling discrimination of subspecies. *Appl. Environ. Microbiol.* 80 4234–4241. 10.1128/AEM.00740-14 24795381 PMC4068689

[B22] Giddens S. R. Houliston G. J. Mahanty H. K. (2003). The influence of antibiotic production and pre-emptive colonization on the population dynamics of *Pantoea agglomerans* (*Erwinia herbicola*) Eh1087 and *Erwinia amylovora* in planta. *Environ. Microbiol.* 5 1016–1021. 10.1046/j.1462-2920.2003.00506.x 14510856

[B23] Glaeser S. P. Kämpfer P. (2015). Multilocus sequence analysis (MLSA) in prokaryotic taxonomy. *Syst. Appl. Microbiol.* 38 237–245. 10.1016/j.syapm.2015.03.007 25959541

[B24] Gomila M. Peña A. Mulet M. Lalucat J. García-Valdés E. (2015). Phylogenomics and systematics in *Pseudomonas*. *Front. Microbiol.* 6:214 10.3389/fmicb.2015.00214 PMC4447124 26074881

[B25] Grissa I. Vergnaud G. Pourcel C. (2007). CRISPRFinder: a web tool to identify clustered regularly interspaced short palindromic repeats. *Nucleic Acids Res.* 35 W52–W57. 10.1093/nar/gkm360 17537822 PMC1933234

[B26] Haas D. Défago G. (2005). Biological control of soil-borne pathogens by fluorescent pseudomonads. *Nat. Rev. Microbiol.* 3 307–319. 10.1038/nrmicro1129 15759041

[B27] Hamdan H. Weller D. M. Thomashow L. S. (1991). Relative importance of fluorescent siderophores and other factors in biological control of *Gaeumannomyces graminis* var. *tritici* by *Pseudomonas fluorescens* 2-79 and M4-80R. *Appl. Environ. Microbiol.* 57 3270–3277. 1838240 10.1128/aem.57.11.3270-3277.1991 PMC183959

[B28] Huerta-Cepas J. Szklarczyk D. Forslund K. Cook H. Heller D. Walter M. C. (2016). eggNOG 4.5: a hierarchical orthology framework with improved functional annotations for eukaryotic, prokaryotic and viral sequences. *Nucleic Acids Res.* 44 D286–D293. 10.1093/nar/gkv1248 26582926 PMC4702882

[B29] Hunt M. Silva N. D. Otto T. D. Parkhill J. Keane J. A. Harris S. R. (2015). Circlator: automated circularization of genome assemblies using long sequencing reads. *Genome Biol.* 16:294. 10.1186/s13059-015-0849-0 26714481 PMC4699355

[B30] Imperiali N. Dennert F. Schneider J. Laessle T. Velatta C. Fesselet M. (2017). Relationships between root pathogen resistance, abundance and expression of pseudomonas antimicrobial genes, and soil properties in representative swiss agricultural soils. *Front. Plant Sci.* 8:427. 10.3389/fpls.2017.00427 28424714 PMC5372754

[B31] Ishimaru C. A. (1988). Multiple antibiotic production by *Erwinia herbicola*. *Phytopathology* 78 746–750. 10.1094/Phyto-78-746

[B32] Jaaffar A. K. M. Parejko J. A. Paulitz T. C. Weller D. M. Thomashow L. S. (2017). Sensitivity of Rhizoctonia isolates to phenazine-1-carboxylic acid and biological control by phenazine-producing *Pseudomonas* spp. *Phytopathology* 107 692–703. 10.1094/PHYTO-07-16-0257-R 28383281

[B33] Johnson K. B. Stockwell V. O. (2000). “Biological control of fire blight,” in *Fire Blight: the Disease and its Causative Agent, Erwinia amylovora* ed. Vanneste J. L. (Wallingford: CABI Publishing) 319–334. 10.1079/9780851992945.0319

[B34] Jones A. L. Schnabel E. L. (2000). “The development of streptomycin-resistant strains of *Erwinia amylovora*,” in *Fire blight: the disease and its causative agent, Erwinia amylovora* ed. Vanneste J. L. (Wallingford: CABI Publishing) 235–251. 10.1079/9780851992945.0235

[B35] Kamber T. Lansdell T. A. Stockwell V. O. Ishimaru C. A. Smits T. H. M. Duffy B. (2012). Characterization of the biosynthetic operon for the antibacterial peptide herbicolin in *Pantoea vagans* biocontrol strain C9-1 and incidence in Pantoea Species. *Appl. Environ. Microbiol.* 78 4412–4419. 10.1128/AEM.07351-11 22504810 PMC3370561

[B36] Keel C. Schnider U. Maurhofer M. Voisard C. Laville J. Burger U. (1992). Suppression of root diseases by *Pseudomonas fluorescens* CHA0: Importance of the bacterial secondary metabolite 24-diacetylphloroglucinol. *Mol. Plant Microbe Interact.* 5 4–13. 10.1094/MPMI-5-004

[B37] King E. O. Ward M. K. Raney D. E. (1954). Two simple media for the demonstration of pyocyanin and fluorescin. *J. Lab. Clin. Med.* 44 301–307. 13184240

[B38] Koren S. Schatz M. C. Walenz B. P. Martin J. Howard J. T. Ganapathy G. (2012). Hybrid error correction and de novo assembly of single-molecule sequencing reads. *Nat. Biotechnol.* 30 693–700. 10.1038/nbt.2280 22750884 PMC3707490

[B39] Llop P. Cabrefiga J. Smits T. H. M. Dreo T. Barbé S. Pulawska J. (2011). *Erwinia amylovora* novel plasmid pEl70: complete sequence, biogeography, and role in aggressiveness in the fire blight phytopathogen. *PLOS ONE* 16:e28651. 10.1371/journal.pone.0028651 22174857 PMC3235134

[B40] Manulis S. Zutra D. Kleitman F. Dror O. David I. Zilberstaine M. (1998). Distribution of streptomycin-resistant strains of *Erwinia amylovora* in Israel and occurrence of blossom blight in the autumn. *Phytoparasitica* 26 223–230. 10.1007/BF02981437

[B41] McGhee G. C. Guasco J. Bellomo L. M. Blumer-Schuette S. E. Shane W. W. Irish-Brown A. (2011). Genetic analysis of streptomycin-resistant (SmR) strains of *Erwinia amylovora* suggests that dissemination of two genotypes Is responsible for the current distribution of SmR *E. amylovora* in Michigan. *Phytopathology* 101 182–191. 10.1094/PHYTO-04-10-0127 20923367

[B42] McManus P. S. Stockwell V. O. Sundin G. W. Jones A. L. (2002). Antibiotic use in plant agriculture. *Annu. Rev. Phytopathol.* 40 443–465. 10.1146/annurev.phyto.40.120301.093927 12147767

[B43] Meyer F. Paarmann D. D’Souza M. Olson R. Glass E. M. Kubal M. (2008). The metagenomics RAST server – a public resource for the automatic phylogenetic and functional analysis of metagenomes. *BMC Bioinformatics* 9:386. 10.1186/1471-2105-9-386 18803844 PMC2563014

[B44] Mikiciński A. Sobiczewski P. Puławska J. Malusa E. (2016). Antagonistic potential of *Pseudomonas* graminis 49M against *Erwinia amylovora*, the causal agent of fire blight. *Arch. Microbiol.* 198 531–539. 10.1007/s00203-016-1207-7 27002332 PMC4930463

[B45] Neilands J. B. (1995). Siderophores: structure and function of microbial iron transport compounds. *J. Biol. Chem.* 270 26723–26726. 10.1074/jbc.270.45.26723 7592901

[B46] Omasits U. Quebatte M. Stekhoven D. J. Fortes C. Roschitzki B. Robinson M. D. (2013). Directed shotgun proteomics guided by saturated RNA-seq identifies a complete expressed prokaryotic proteome. *Genome Res.* 23 1916–1927. 10.1101/gr.151035.112 23878158 PMC3814891

[B47] Omasits U. Varadarajan A. R. Schmid M. Goetze S. Melidis D. Bourqui M. (2017). An integrative strategy to identify the entire protein coding potential of prokaryotic genomes by proteogenomics. *Genome Res.* 27 2083–2095. 10.1101/153213 29141959 PMC5741054

[B48] Parejko J. A. Mavrodi D. V. Mavrodi O. V. Weller D. M. Thomashow L. S. (2013). Taxonomy and distribution of phenazine-1-carboxylic acid-producing *Pseudomonas* spp. in the dryland agroecosystem of the Inland Pacific Northwest. *Appl. Environ. Microbiol.* 79 3887–3891. 10.1128/AEM.03945-12 23584779 PMC3675913

[B49] Paternoster T. Défago G. Duffy B. Gessler C. Pertot I. (2010). Selection of a biocontrol agent based on a potential mechanism of action: degradation of nicotinic acid, a growth factor essential for *Erwinia amylovora*. *Int. Microbiol.* 13 195–206. 21404214 10.2436/20.1501.01.126

[B50] Paulin J. P. Samson R. (1973). Le feu bactérien en France. II.caractères des souches d’*Erwinia amylovora* (Burril) Winslow et al. 1920 isolées du foyer franco-belge. *Annal. Phytopathol.* 5 389–397.

[B51] Pierson L. S. III Keppenne V. D. Wood D. W. (1994). Phenazine antibiotic biosynthesis in *Pseudomonas aureofaciens* 30-84 is regulated by PhzR in response to cell density. *J. Bacteriol.* 176 3966–3974. 10.1128/jb.176.13.3966-3974.1994 8021179 PMC205594

[B52] Pujol M. Badosa E. Cabrefiga J. Montesinos E. (2005). Development of a strain-specific quantitative method for monitoring *Pseudomonas fluorescens* EPS62e, a novel biocontrol agent of fire blight. *FEMS Microbiol.* 249 343–352. 10.1016/j.femsle.2005.06.029 16006071

[B53] Pusey P. L. (1997). Crab apple blossoms as a model for research on biological control of fire blight. *Phytopathology* 87 1096–1102. 10.1094/PHYTO.1997.87.11.1096 18945005

[B54] Pusey P. L. Rudell D. R. Curry E. A. Mattheis J. P. (2008a). Characterization of stigma exudates in aqueous extracts from apple and pear flowers. *HortScience* 43 1471–1478.

[B55] Pusey P. L. Stockwell V. O. Reardon C. L. Smits T. H. M. Duffy B. (2011). Antibiosis activity of *Pantoea agglomerans* biocontrol strain E325 against *Erwinia amylovora* on apple flower stigmas. *Phytopathology* 101 1234–1241. 10.1094/PHYTO-09-10-0253 21679036

[B56] Pusey P. L. Stockwell V. O. Rudell D. R. (2008b). Antibiosis and acidification by *Pantoea agglomerans* strain E325 may contribute to suppression of *Erwinia amylovora*. *Phytopathology* 98 1136–1143. 10.1094/PHYTO-98-10-1136 18943460

[B57] Remus-Emsermann M. N. P. Schmid M. Gekenidis M.-T. Pelludat C. Frey J. E. Ahrens C. H. (2016). Complete genome sequence of *Pseudomonas citronellolis* P3B5 a candidate for microbial phyllo-remediation of hydrocarbon-contaminated sites. *Stand. Genomic Sci.* 11:75. 10.1186/s40793-016-0190-6 28300228 PMC5037603

[B58] Rosendahl C. N. Olson L. W. (1992). An in vivo screening method for antifungal activity against the plant pathogen *Pythium ultimum* Trow. *J. Phytopathol.* 134 324–328. 10.1111/j.1439-0434.1992.tb01240.x

[B59] Schwyn B. Neilands J. B. (1987). Universal chemical assay for the detection and determination of siderophores. *Anal. Biochem.* 160 47–56. 10.1016/0003-2697(87)90612-9 2952030

[B60] Seibold A. Fried A. Kunz S. Moltmann E. Lange E. Jelkmann W. (2004). Yeasts as antagonists against fireblight. *Eppo Bull.* 34 389–390. 10.1111/j.1365-2338.2004.00766.x

[B61] Silby M. W. Winstanley C. Godfrey S. A. C. Levy S. B. Jackson R. W. (2011). *Pseudomonas* genomes: diverse and adaptable. *FEMS Microbiol. Rev.* 35 652–680. 10.1111/j.1574-6976.2011.00269.x 21361996

[B62] Stamatakis A. (2014). RAxML version 8: a tool for phylogenetic analysis and post-analysis of large phylogenies. *Bioinformatics* 30 1312–1313. 10.1093/bioinformatics/btu033 24451623 PMC3998144

[B63] Stockwell V. O. Johnson K. B. Sugar D. Loper J. E. (2002). Antibiosis contributes to biological control of fire blight by *Pantoea agglomerans* strain Eh252 in orchards. *Phytopathology* 92 1202–1209. 10.1094/PHYTO.2002.92.11.1202 18944246

[B64] Stothard P. Wishart D. S. (2005). Circular genome visualization and exploration using CGView. *Bioinformatics* 21 537–539. 10.1093/bioinformatics/bti054 15479716

[B65] Stutz E. W. Defago G. Kern H. (1986). Naturally occurring fluorescent pseudomonads involved in suppression of black root-rot of tobacco. *Phytopathology* 76 181–185. 10.1094/Phyto-76-181

[B66] Takikawa Y. Serizawa S. Ichikawa T. Tsuyumu S. Goto M. (1989). *Pseudomonas syringae* pv. *actinidiae* pv. nov.: the causal bacterium of canker of kiwifruit in Japan. *Jpn. J. Phytopathol.* 55 437–444. 10.1094/PHYTO-03-12-0064-R 22877312

[B67] Tatusova T. DiCuccio M. Badretdin A. Chetvernin V. Nawrocki E. P. Zaslavsky L. (2016). NCBI prokaryotic genome annotation pipeline. *Nucleic Acids Res.* 44 6614–6624. 10.1093/nar/gkw569 27342282 PMC5001611

[B68] Thomashow L. S. Weller D. M. Bonsall R. F. Pierson L. S. (1990). Production of the antibiotic phenazine-1-carboxylic acid by fluorescent *Pseudomonas* species in the rhizosphere of wheat. *Appl. Environ. Microbiol.* 56 908–912. 16348176 10.1128/aem.56.4.908-912.1990 PMC184320

[B69] Thomson S. V. (2000). “Epidemiology of fire blight,” in *Fire Blight: the Disease and its Causative Agent, Erwinia amylovora* ed. Vanneste J. L. (Wallingford: CABI Publishing) 9–36. 10.1079/9780851992945.0009

[B70] Velasco A. Acebo P. Gomezyu A. Schleissner C. Rodríguez P. Aparicio T. (2005). Molecular characterization of the safracin biosynthetic pathway from *Pseudomonas fluorescens* A2-2: designing new cytotoxic compounds. *Mol. Microbiol.* 56 144–154. 10.1111/j.1365-2958.2004.04433.x 15773985

[B71] Wilson M. Lindow S. E. (1993). Interaction between the biological control agent *Pseudomonas fluorescens* A506 and *Erwinia amylovora* in pear blossoms. *Phytopathology* 83 117–123. 10.1094/Phyto-83-117

[B72] Wright S. A. I. Zumoff C. H. Schneider L. Beer S. V. (2001). *Pantoea agglomerans* strain EH318 produces two antibiotics that inhibit *Erwinia amylovora* in vitro. *Appl. Environ. Microbiol.* 67 284–292. 10.1128/AEM.67.1.284-292.2001 11133457 PMC92566

[B73] Young J. M. (1988). *Pseudomonas syringae* pv. *persicae* from nectarine, peach, and Japanese plum in New Zealand. *Bull. OEPP* 18 141–151. 10.1111/j.1365-2338.1988.tb00359.x

