# Supplementary material for: Pseudomonas orientalis F9: A Potent Antagonist against Phytopathogens with Phytotoxic Effect in the Apple Flower
Source: Front Microbiol. 2018 Feb 9;9:145. doi: 10.3389/fmicb.2018.00145 (PMC5811506; doi:10.3389/fmicb.2018.00145)
Supplement: Supplementary file 1 [file Table_1.DOCX]

Supplementary Table 1. Number of genes associated with general COG functional categories.

The total is based on the total number of predicted protein coding genes in the genome.

| **Code** | **Value** | **%age** | **Description** |
| --- | --- | --- | --- |
| J | 175 | 3.40 | Translation, ribosomal structure and biogenesis |
| A | 1 | 0.02 | RNA processing and modification |
| K | 401 | 7.80 | Transcription |
| L | 163 | 3.17 | Replication, recombination and repair |
| B | 3 | 0.06 | Chromatin structure and dynamics |
| D | 37 | 0.72 | Cell cycle control, Cell division, chromosome partitioning |
| V | 61 | 1.19 | Defense mechanisms |
| T | 254 | 4.94 | Signal transduction mechanisms |
| M | 272 | 5.29 | Cell wall/membrane biogenesis |
| N | 75 | 1.46 | Cell motility |
| U | 84 | 1.63 | Intracellular trafficking and secretion |
| O | 167 | 3.25 | Posttranslational modification, protein turnover, chaperones |
| C | 256 | 4.98 | Energy production and conversion |
| G | 231 | 4.49 | Carbohydrate transport and metabolism |
| E | 414 | 8.05 | Amino acid transport and metabolism |
| F | 108 | 2.10 | Nucleotide transport and metabolism |
| H | 150 | 2.92 | Coenzyme transport and metabolism |
| I | 159 | 3.09 | Lipid transport and metabolism |
| P | 291 | 5.66 | Inorganic ion transport and metabolism |
| Q | 100 | 1.94 | Secondary metabolites biosynthesis, transport and catabolism |
| R | 0 | 0.00 | General function prediction only |
| S | 1297 | 25.22 | Function unknown |
| - | 443 | 8.62 | Not in COGs |
|  | **5142** | **100.00** | **Total** |
