# Supplementary material for: Pseudomonas orientalis F9: A Potent Antagonist against Phytopathogens with Phytotoxic Effect in the Apple Flower
Source: Front Microbiol. 2018 Feb 9;9:145. doi: 10.3389/fmicb.2018.00145 (PMC5811506; doi:10.3389/fmicb.2018.00145)
Supplement: Supplementary file 2 [file Table_2.DOCX]

Supplementary Table 2. A comparison of the assembly level of the two already available *P. orientalis* genome assemblies versus the *de novo* assembly of strain F9.

| ***P. orientalis* strain** | **Genome size [Mb]** | **# of contigs** | **# of N's** | **Gaps** | **NCBI "Assembly level"** | **Sequencing technology** | **Database** |
| --- | --- | --- | --- | --- | --- | --- | --- |
| F9 | 5.98 | 1 | 0 | 0 | "Complete" (Means: complete sequence & circularized) | PacBio | GenBank |
| BS2775 | 6.13 | 1 | 100 | 1 gap | "Chromosome" (Means: potentially non complete sequence & not circularized) | PacBio | RefSeq |
| DSM 17489 | 6.19 | 39 | 0 | >= 39 gaps | "Contig" (Means: assembly is fragmented and parts of the genome are likely missing) | MiSeq | RefSeq |
