# Supplementary material for: Pseudomonas orientalis F9: A Potent Antagonist against Phytopathogens with Phytotoxic Effect in the Apple Flower
Source: Front Microbiol. 2018 Feb 9;9:145. doi: 10.3389/fmicb.2018.00145 (PMC5811506; doi:10.3389/fmicb.2018.00145)
Supplement: Supplementary file 3 [file Table_3.DOCX]

Supplementary Table 3. NCBI Accessions of strains used for construction of the phylogenetic tree.

| **Species** | **Strain** | **NCBI Accession** |
| --- | --- | --- |
| *Pantoea vagans* | C9-1 | NC_014562 |
| *Pseudomonas cedrina* | BS2981 | LT629753 |
| *Pseudomonas citronellolis* | P3B5 | NZ_CP014158 |
| *Pseudomonas extremorientalis* | BS2774 | LT629708 |
| *Pseudomonas fluorescens* | A506 | NC_017911 |
| *Pseudomonas fluorescens* | SBW25 | NC_012660 |
| *Pseudomonas graminis* | UASWS1507 | NZ_MDEN01000000 |
| *Pseudomonas libanensis* | BS2975 | LT629699 |
| *Pseudomonas marginalis* | ICMP 3553 | NZ_LKEG01000000 |
| *Pseudomonas orientalis* | F9 | CP018049 |
| *Pseudomonas orientalis* | BS2775* | LT629782 |
| *Pseudomonas orientalis* | DSM 17489* | NZ_JYLM01000000 |
| *Pseudomonas syringae pv. actinidiae* | ICMP 9617 | AOKP01000000 |
| *Pseudomonas syringae pv. persicae* | NCPPB 2254 | NZ_LAZV01000000 |

* The genome sequences of the two available *P. orientalis* strains are not

closed/complete according to NCBI
