# Supplementary material for: Pseudomonas orientalis F9: A Potent Antagonist against Phytopathogens with Phytotoxic Effect in the Apple Flower
Source: Front Microbiol. 2018 Feb 9;9:145. doi: 10.3389/fmicb.2018.00145 (PMC5811506; doi:10.3389/fmicb.2018.00145)
Supplement: Supplementary file 4 [file Table_4.DOCX]

Supplementary Table 4. Positions of putative genomic islands detected using

IslandViewer 3 (overlapping intervals are not collapsed).

| **Start (pos. on genome)** | **End (pos. on genome)** | **Length (bp)** |
| --- | --- | --- |
| 195229 | 209952 | 14723 |
| 219394 | 223709 | 4315 |
| 820200 | 824623 | 4423 |
| 885951 | 890252 | 4301 |
| 1142086 | 1147250 | 5164 |
| 1590954 | 1600668 | 9714 |
| 1593538 | 1608054 | 14516 |
| 1602749 | 1645511 | 42762 |
| 1608071 | 1646103 | 38032 |
| 1795834 | 1811367 | 15533 |
| 2176411 | 2180915 | 4504 |
| 2700412 | 2705529 | 5117 |
| 2750205 | 2754869 | 4664 |
| 2774813 | 2783299 | 8486 |
| 2842860 | 2858434 | 15574 |
| 2863004 | 2867330 | 4326 |
| 2880626 | 2908019 | 27393 |
| 2942106 | 2947170 | 5064 |
| 2942313 | 2947124 | 4811 |
| 3003283 | 3010523 | 7240 |
| 3003560 | 3011598 | 8038 |
| 3021215 | 3032479 | 11264 |
| 3065147 | 3069497 | 4350 |
| 3094936 | 3099186 | 4250 |
| 3109227 | 3116219 | 6992 |
| 3121997 | 3127505 | 5508 |
| 3136238 | 3140869 | 4631 |
| 3211778 | 3218282 | 6504 |
| 3460450 | 3468644 | 8194 |
| 3495460 | 3500339 | 4879 |
| 3500741 | 3508824 | 8083 |
| 3501958 | 3512084 | 10126 |
| 3534477 | 3546174 | 11697 |
| 3776549 | 3787009 | 10460 |
| 3814391 | 3820544 | 6153 |
| 3820926 | 3825209 | 4283 |
| 3856587 | 3864415 | 7828 |
| 3942343 | 3958007 | 15664 |
| 3949107 | 3953538 | 4431 |
| 3996056 | 4008123 | 12067 |
| 4008140 | 4021852 | 13712 |
| 4016424 | 4042519 | 26095 |
| 4017307 | 4041632 | 24325 |
| 4685615 | 4693275 | 7660 |
| 4685939 | 4690546 | 4607 |
| 4905942 | 4927564 | 21622 |
| 4905942 | 4910094 | 4152 |
| 4912658 | 4923018 | 10360 |
| 4914011 | 4927092 | 13081 |
| 5408819 | 5423180 | 14361 |
| 5413643 | 5422058 | 8415 |
| 5771183 | 5781775 | 10592 |
| 5956262 | 5961307 | 5045 |
